# Supporting efficiency improvement in public health systems: a rapid evidence synthesis

**DOI:** 10.1186/s12913-022-07694-z

**Published:** 2022-03-03

**Authors:** James Kenneth Walters, Anurag Sharma, Emma Malica, Reema Harrison

**Affiliations:** 1 New South Wales Ministry of Health, St Leonards, Australia; 2 UNSW Sydney, Sydney, Australia; 3 Macquarie University, Sydney, Australia

## Abstract

**Background:**

Public health systems internationally are under pressure to meet increasing demand for healthcare in the context of increasing financial resource constraint. There is therefore a need to maximise health outcomes achieved with public healthcare expenditure. This paper aims to establish and synthesize the contemporary evidence base for approaches taken at a system management level to improve efficiency.

**Methods:**

Rapid Evidence Assessment (REA) methodology was employed. A search strategy was developed and applied (PUBMED, MEDLINE) returning 5,377 unique titles. 172 full-text articles were screened to determine relevance with 82 publications included in the final review. Data regarding country, study design, key findings and approaches to efficiency improvement were extracted and a narrative synthesis produced. Publications covering health systems from developed countries were included.

**Results:**

Identified study designs included policy reviews, qualitative reviews, mixed methods reviews, systematic reviews, literature reviews, retrospective analyses, scoping reviews, narrative papers, regression analyses and opinion papers. While findings revealed no comprehensive frameworks for system-wide efficiency improvement, a range of specific centrally led improvement approaches were identified. Elements associated with success in current approaches included dedicated central functions to drive system-wide efficiency improvement, managing efficiency in tandem with quality and value, and inclusive stakeholder engagement.

**Conclusions:**

The requirement for public health systems to improve efficiency is likely to continue to increase. Reactive cost-cutting measures and short-term initiatives aimed only at reducing expenditure are unlikely to deliver sustainable efficiency improvement. By providing dedicated central system-wide efficiency improvement support, public health system management entities can deliver improved financial, health service and stakeholder outcomes.

**Supplementary Information:**

The online version contains supplementary material available at 10.1186/s12913-022-07694-z.

## Introduction

Public health systems, being those providing publicly-funded healthcare, are under intense pressure to meet increasing demand for health care in environments of considerable and increasing financial resource constraint [1]. Pressures that pervade health systems internationally include tightening budgets, growing demand, increasing disease burdens, increasing burden on infrastructure, technological advancements, changing service models, increasing consumer expectations and changing service accessibility [2]. An ongoing state of inefficiency amidst an environment of seemingly-constant reform is therefore characteristic of the public health systems of many well-developed countries around the world [3, 4]. Compounding this is a common trend in rates of growth in health services expenditure in excess of funding growth rates, creating further pressure on already-burdened public health systems [5, 6].

Although the models through which public health systems are funded and structured vary across developed nations, the function of Government in establishing health system budgets and setting service requirements is a common principle across developed countries [7]. Public health systems are then administered centrally by system management entities such as Ministries of Health, Departments of Health, District Boards and Trusts [7]. Within the focus on value-based healthcare over the past decade, efficiency is seen as a key component of value in healthcare and is defined as maximizing health outcomes achieved for resources invested while also maintaining quality, safety and experience outcomes [8, 9].

Ongoing and common challenges to efficiency are faced despite variation in system structures, well-established system performance indicators and the existence of longstanding literature on efficiency measurement in health contexts. These challenges include achieving sustainable expenditure levels, reducing inefficiency and reducing waste [10]. Studies focusing on concurrently addressing these challenges at a system-wide level are rare. This review finds that although contemporary evidence for approaches to supporting efficiency improvement in public health systems lacks common consensus on a single standard of best practice, a range of examples where such support has been successfully managed are evident across the literature.

There is therefore an urgent need to consolidate understanding of contemporary evidence-based approaches for management bodies of public health systems to support efficiency improvement across the health systems they administrate. To address the identified knowledge gap, a rapid evidence synthesis was undertaken to consolidate contemporary research. The following review questions were addressed: 1) How is efficiency improvement conceptualised in public health systems? And 2) How do public health system management bodies support efficiency improvement across their health systems? Question one was approached first in order to set the context for the paper, with question two being the primary focus of the review.

## Methods

Rapid Evidence Assessment (REA), an established approach in public health policy and management research for exploring broad and complex issues, was used to address the review questions [11, 12]. This model rigorously follows established systematic review methodology to search and appraise existing evidence, limiting selected aspects of the review process to shorten the review timespan while still enabling the depth of current knowledge to be appraised [13–15]. The primary rationale for selecting the REA approach for this study was due to the nature of the body of literature, rather than a shorter timeframe. The REA approach was well-suited to this topic due to the disparate nature of the evidence base [16]. This study followed the CEBMa guideline for Rapid Evidence Assessments, with REA methodology applied to limit the review by searching only two major relevant databases, extracting only the study findings specifically relevant to answering the review questions and excluding grey literature [13, 17]. A review protocol was developed to guide the study process shown below, establishing eligibility criteria, exclusion criteria, search strategy and to guide data extraction and synthesis as outlined below. The review protocol was not registered due to the study timeframe.

## Eligibility criteria

Full-text publications available in English relating to public health systems in developed countries were included. A timeframe for inclusion of papers published in or after 2011 was established to identify contemporary peer-reviewed evidence. Grey literature was excluded. Papers not available in English were excluded from the study, as were title matches for which full papers were not available. Papers based in developing countries were excluded. Papers which held no clear content relating to the role and functions of central public health system management entities in efficiency improvement were excluded. Papers specific to COVID-19 were also excluded.

## Search strategy

A range of text words, synonyms and subject headings were developed for the major concepts of health system, efficiency and improvement. These text words, synonyms and subject headings were used to undertake a systematic search of two electronic databases that index journals of relevance to the review topic (MEDLINE, PUBMED) from January 2011 to February 2019. This search was updated in May 2021. Results were merged using reference-management software (Endnote, version X9) and duplicates removed with the review process managed using Covidence software.

## Study selection and data extraction

Two reviewers (JW, RH) independently screened titles and abstracts for potentially relevant articles. Full text articles were obtained and eligibility criteria independently applied by the two reviewers, with a third reviewer (AS) engaged to resolve conflicts and provide a face validity check. One author (JW) extracted the data, with extracted data periodically checked by a second reviewer (RH) throughout the extraction process. The following data were extracted: first author, publication year, country, study objective, sample, methods, key study findings and approaches to efficiency improvement.

## Data synthesis

Based on the study objectives, findings were synthesised in a narrative empirical synthesis [18]. A quantitative synthesis was not appropriate due to heterogenicity of the study design. Initial descriptions of the eligible studies and results were tabulated and patterns in the data explored to identify consistent findings in relation to the review objectives. Interrogation of the findings explored relationships between publication characteristics and their findings; the data emerging from different publications; and the influence of context on emergent findings.

## Results

**Fig. 1:**
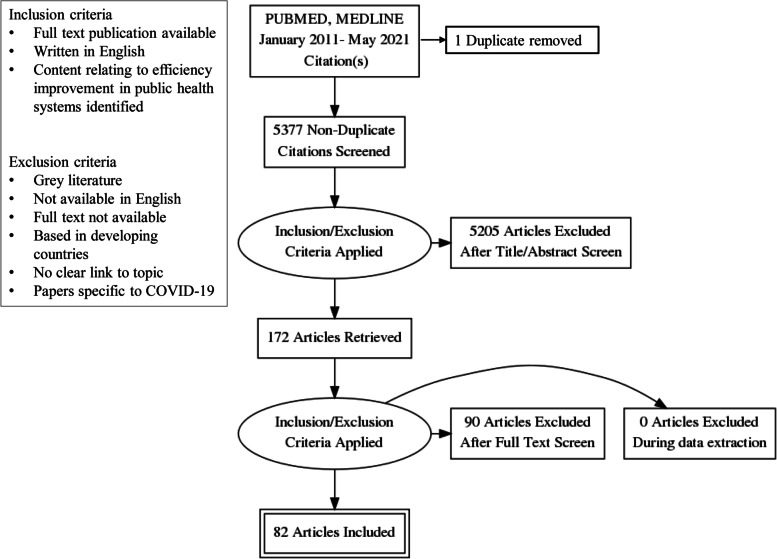
PRISMA diagram

The two database searches returned a result of 5,377 unique titles, with 172 publications selected for full review and 82 papers included in the study. Results were reported using the PRISMA statement as shown in Fig. 1 below [19]:

## Review findings

11 studies were conducted in Australia, 22 in the United States of America and 15 in international settings. The remaining 34 studies were broadly distributed across a range of developed nations. Most studies were published around the midpoint of the study period, with 52 of 82 studies published between 2014 and 2018 and only nine studies published after 2018. Study designs included 13 mixed methods approaches, 12 retrospective studies, 11 narrative syntheses, 8 qualitative studies, 6 systematic reviews and 4 literature reports.

Financial sustainability concerns regarding public health systems in developed countries were widely acknowledged, along with the associated need for efficiency improvement to meet these concerns. Included publications described various approaches to supporting efficiency improvement, however no single, best-practice or evidence-based comprehensive framework for managing efficiency at the system level was identified. The wide range of countries in which studies were set and the differences between political and health system structures within them may have influenced the variety seen in efficiency improvement approaches.

### How is efficiency improvement conceptualised in public health systems?

Efficiency in health services is well-defined in the literature, with clear links to expenditure and health service outcomes. Efficiency can be considered in technical and allocative terms within the context of existing resources, operating requirements, regulatory environments and health service outputs [20, 21]. Productive efficiency involves making the most of available resources while maximizing outputs [1]. It was widely agreed that in healthcare these concepts extend to the delivery of services with comprehensiveness, coordination, accessibility, quality and continuity [22]. The concepts of efficiency and value were commonly associated with weighing outcomes against the costs required to achieve them [23, 24]. Within the current paradigm of value-based health services, financial efficiency and outcomes achieved for inputs invested are considered core elements of value [3, 24].

### How do public health system central management entities support efficiency improvement across their health systems?

Three strategic aims emerged: the role of the system management entity in leading and enabling efficiency improvement across the healthcare system, concurrently managing financial and health service outcomes, and stakeholder considerations relevant for system management entities when developing and implementing approaches to efficiency improvement. A summary of system-wide efficiency improvement approaches identified in this review is presented in Table 1 below.

**Table 1:** Summary of system-wide efficiency improvement approaches

Strategic aim	Specific strategies identified	Source
Centralised efficiency improvement management	Utilise peer performance benchmarking to identify and share high-performing services, models, initiatives and approaches	Allin, Grignon & Wang 2016 [1] Grimes et al. 2011 [25] Nuti et al. 2016 [25] Rumbhold et al. 2015 [23] White, 2015 [26] Alatawi, Niessen & Khan 2020 [27]
	Establish service level agreements and performance targets to set expectations and delivery accountabilities	Anderson & Catchlove 2012 [28] Christiansen & Vrangbaek 2018 [29] Zhang, Tone & Lu 2018 [30]
	Sponsor efficiency practice networks for system-wide collaboration and knowledge-sharing	Auerbach et al. 2014 [31]
	Provide financial incentives for adoption of efficient practices	Bradford et al. 2016 [32] Elshaug et al. 2017 [33]
	Establish system-wide policy guidance on which practices are most efficient and which lower-value practices should be replaced	Elshaug et al. 2017 [33] Garcia-Armesto, Campillo-Artero & Bernal-Delgado 2013
	Establish best practice guidance for financial and management decision-makers on resource allocation and reallocation	Elshaug et al. 2017 [33] Harris et al. 2017 [34]
	Establish a centralised efficiency improvement unit to support system-wide improvement processes	Hassanian, 2017 [2] Lavoie-Tremblay et al. 2012 [35]
	Address reducing duplication and maximising asset utilisation at a whole-of-system level	Pencheon, 2015 [36] Tsai et al. 2017 [37]
	Ensure timely, transparent performance reporting for improvement initiatives	Tataw, 2014 [38] Alatawi, Niessen & Khan 2020 [27]
Concurrently improving efficiency, quality and value	Establish system-wide policy for balancing expenditure, quality and value	Akinleye et al. 2019 [39]
	Integrate financial, workforce and clinical service data to model improvement impact	Birch et al. 2015 [40]
	Systematically identify and address health service overuse/underuse	Ellen et al. 2018 [6] Elshaug et al. 2017 [33] Kumar 2011 [41]
	Partner with primary care services to enable early intervention	Fiorentini et al. 2011 [42] Gaertner, Maier & Radbruch 2015 [43]
	Determine a sufficient time period within which efficiency improvement initiatives can be delivered and realised	Hebert et al. 2014 [44] Schakel, Wu & Jeurissen 2018 [45]
	Weigh costs of innovation with potential efficiency and value generated	Mussap, 2014 [46]
Engaging stakeholders in efficiency improvement	Include frontline staff and managers in designing efficiency improvement initiatives	Ashton, Bramley & Armstrong 2012 [47] De Rosis & Nuti 2018 [48] Elshaug et al. 2017 [33]
	Leverage evidence of combined cost and patient outcome improvements to promote stakeholder acceptance of efficiency approaches	Gans et al. 2012 [49] Murphy et al. 2016 [50]
	Link frontline staff performance goals with organisational improvement goals	Kämäräinen et al. 2016 [51]
	Continue to engage with improvement initiative stakeholders following implementation to promote improvement longevity	Lennox, Maher & Reed 2018 [52]
	Establish clear and transparent improvement targets at the health service level	Nuti et al. 2016 [53] Moberg & Fredrikkson 2020 [54] Christiansen & Vrangbaek 2018
	Tailor resource allocation and service optimisation messaging to promote frontline clinician and management engagement	Moberg & Fredriksson 2020 [54] Harris et al. 2017 [34] Wolfenden et al. 2019 [55]

#### Central support and leadership for system-wide efficiency improvement

Increasing resource pressure and demand are heightening the focus on efficiency across public health systems [36]. Specific, clear, centralized and coordinated support for efficiency improvement initiatives is critical to improved efficiency at a system-wide level [42]. Despite these factors, dedicated resources and policy guidelines for enhancing public health service efficiency are often limited [1, 56]. Across the literature, examples of facility-level efficiency improvement projects were common however studies focusing on efficiency improvement at the system level were rare [1].

Studies identified that efficiency levels are variable within as well as between systems, and that the decentralized, department-based nature of healthcare facilities is a potential source of inefficiency [57, 58]. These studies suggested that reform is commonly employed in response, and that while reform can drive short term efficiency change, long-term financial sustainability requires ongoing focus and monitoring [59]. It was further suggested that reform intended to improve financial performance through “one size fits all” approaches did not improve efficiency, while establishing central guidance for desired service outcomes and priority areas for resource allocation were effective in achieving both efficiency and quality improvements [28, 30, 60].

It was suggested that efficiency can be centrally promoted across decentralized systems through the establishment of system-wide priority setting, training, policy frameworks and business models [58, 61]. It was widely reported that agreements and performance measures or indicators are established system management tools across devolved governance models, however there was no clear consensus on the use of efficiency improvement measures within these broader processes [28, 62]. Alignment between efficiency and quality performance indicators along with transparency in target setting and performance reporting was linked with successful efficiency improvement programs [53, 63].

Two studies found that evaluation of efficiency improvement is complicated by initiatives which take several years to produce favorable impacts, especially with regard to annual financial cycles [44, 64]. Financial rule-setting has been associated with a modest reduction in public health expenditure, however this may not occur until 1–2 years following implementation [45]. Realistic timeframes of up to three years for efficiency improvement must therefore be considered [44]. Establishing prompts, targets, guidelines, triggers and mandatory requirements for resource allocation consideration was linked with successfully embedding efficiency improvement and disinvestment practices [65]. This can also be supported through identifying points where efficiencies are redistributed throughout the system [32]. It was proposed that focusing on productivity, savings, waste reduction and resource maximization together can promote efficiency and quality outcomes concurrently [34, 47]. One study suggested that productivity is less frequently used as a measure of efficiency than other efficiency-related measures [37].

Studies included in the review consistently identified the use of benchmarking and data use as key enablers of efficiency improvement. Local networking and benchmarking can promote collaboration by identifying high-performing and low-performing facilities, however consideration to variations in cost and complexity between individual services was advised [53, 66, 67]. Peer benchmarking was proposed as an enabler for improvement through the identification of approaches which are achieving positive outcomes for sharing with similar settings elsewhere [25, 26, 68]. It was suggested that this promotes stakeholder acceptance through increasing buy-in and building evidence of success [69]. The ability to adapt an initiative to suit local needs, including modification over time was noted as a key long-term success factor [69]. Consideration to timely reporting was recommended as delays in central performance reporting were perceived as a barrier to engagement and performance management [69–71].

The longevity of efficiency improvements is not guaranteed upon project completion, with one study suggesting that only 60% of improvement projects in health are able to maintain at least one key project element on an ongoing basis [52]. This finding was accompanied by a call for a system-wide focus on embedding key improvement elements upon project completion. Where improvement projects had failed, it was also recommended to identify causes of failure and take action to ensure failures were not repeated [72].

One series of papers focused on reallocating resources away from outdated or inefficient processes and infrastructure, a process framed as disinvestment [34]. This series identified that sharing risks, goals, responsibilities and feedback across public health systems can promote a sustainable win–win outcome for stakeholders and public health systems [48]. Health systems can utilize performance and activity data to identify opportunities for disinvestment, track progress towards targets and evaluate the impact of initiatives [73, 74]. It was proposed that disinvestment and investment should be considered in parallel rather than separately [75]. This series also suggested that planned improvements to services and processes must fit with existing structures in order to be sustained [69].

Several factors associated with efficiency were identified in only one or two studies. System-wide guidance in identifying low-value and high-risk procedures, removing less efficient choices, producing policy guidelines, embedding regulatory frameworks for cost-effective alternatives and raising the profile of high and low-value intervention was recommended [71, 76]. The identification and scaling-up of high-value initiatives such as service over-use and under-use was identified as an area of opportunity [77]. Evidence for cost savings in similar initiatives elsewhere could be used to justify investment decisions and develop business cases [43]. One study proposed that centralizing and consolidating services can enhance performance, as can centrally set efficiency targets [29]. Evidence was mixed for extending this approach to public–private partnerships, which were described as complex, difficult to implement and dependent upon a range of pre-existing conditions to succeed [78, 79]. Pay-for-quality schemes and mergers between public hospitals were flagged as similarly fraught with limited evidence for improvement [80, 81].

Four studies presented evidence that centralized efficiency improvement units can have an impact on improving efficiency across public health systems by providing support and reducing pressure on frontline managers [35]. Support adoption was identified as a key challenge, often influenced by the engagement and enthusiasm of senior leadership [2]. These studies found that the decision-making level of the efficiency improvement unit has significant bearing on the focus and activities of the unit [1]. Evidence identified for the potential impact of central efficiency improvement support functions suggested that these functions solve operational issues, reduce spending, share expertise, standardize practices, build capability, provide progress reporting, promote communication, assist delivery, optimize processes, evaluate impacts and promote stakeholder satisfaction [35].

Challenges to such units included task prioritization, managing expectations and managing multiple concurrent projects [35]. Stakeholder perceptions of the capabilities and knowledge of these teams was a determinant of engagement [2]. It was suggested that appointing team members recognized for experience, credibility and familiarity would facilitate support uptake. Effective management of stakeholder expectations was also linked to the effectiveness of such teams [35].

#### Concurrently managing efficiency, delivery and quality outcomes

Included studies identified a number of recent unsuccessful central approaches to improving financial performance such as “cost containment” initiatives, with limited consideration of impacts on care quality and potential longer-term impacts to service delivery [30, 80]. Over-restructuring, focusing on short-term goals, short-term funding approaches and cultures of complacency were also identified as barriers [55, 82]. Inadequate or insufficient business plans were associated with risk of unsuccessful improvement delivery [83]. Activities that may not represent genuine efficiency improvement were also apparent, such as increasing coding acuity and coding creep to attract greater activity-based funding and selective picking of high-value activity [84, 85].

Links between care quality, care outcomes and the investments required to achieve them are well-embedded in models of health care efficiency [86]. As such, approaches that improve both efficiency and health outcomes concurrently are required, with support for this evident across papers describing the shift towards value-based health care [87]. It was proposed that this emerging focus on concurrently improving both quality and efficiency replaces the previous focus on purely financial efficiency, with evidence suggesting that focusing solely on financial efficiency does not result in health service efficiency improvement [30]. Further evidence indicated that strong financial performance is associated with higher service quality and patient satisfaction [39].

At a system level the considerations required to support efficiency improvement included establishing a system-wide focus, addressing unwarranted clinical variation, appropriate funding levers and monitoring performance indicators for efficiency, service outcomes and satisfaction [23, 49]. Studies noted that implementation can be successfully approached through setting policy levers which prioritize affordability, access to care and innovation rather than targeting price or utilization-based metrics as priority outcomes [27, 39, 88].

Inefficiency in public health systems cannot be rectified simply through additional resourcing [3]. It was generally accepted that quality, cost and value are interlinked rather than isolated [3, 41, 68]. Addressing overuse and low-value activities was shown to improve both service effectiveness and efficiency [6]. Importantly, health services cannot be expected to operate at near-maximum efficiency as this impacts capacity for workforce training, research and innovation [89]. This calls for the setting of realistic and balanced efficiency improvement targets, management solutions and timeframes. It was further suggested that efficiency improvement training, consistent leadership and continued post-implementation monitoring are required in order for improvements to be sustained [2].

#### Stakeholder engagement

This review consistently found that underpinning any efficiency improvement initiative are concurrent requirements to improve patient and staff experience, therefore stakeholder engagement is key [49]. Addressing staff resistance and stakeholder expectations were noted as significant challenges for efficiency programs with common misconceptions amongst staff and patients that "more is better" and "newer is better" [6]. When engaging stakeholders in efficiency improvement processes, the terms “optimization” and “resource allocation” were proposed as more agreeable terms than discontinuation and reduction [55, 63]. The risk of resource investment being considered a waste if no value is added to patient outcomes was apparent [48]. To mitigate this risk, the involvement of stakeholders in target-setting was associated with improved acceptance and compliance towards efficiency targets [90]. Clear target definition and stakeholder collaboration during improvement initiative development were reported as factors for improvement initiative success [50, 91].

Collaboration, knowledge sharing and performance monitoring were associated with project impact and sustainable improvements [2]. It was clear throughout the studies reviewed that effective, ongoing consultation and collaboration is a critical element in efficiency improvement [48]. Prioritizing these principles at a whole-of-system level through policy and strategic priorities can enable consistent system-wide approaches to stakeholder engagement. This included effective consultation and collaboration with leaders from across the healthcare system in addition to those within the central system management body [54]. Despite increasing trends to involve the public in healthcare system decision making and priority-setting, evidence for the impact of public involvement in efficiency improvement processes or development programs to enable public involvement was extremely limited [92–94].

The reviewed studies indicated that stakeholders are unlikely to be motivated to engage in efficiency improvement solely because of cost-effectiveness [91]. Linking unit manager and organizational goals, measures and incentives was found to promote system-wide efficiencies [51]. System management entities can sponsor collaborative networks to share successful practice and performance information, enabled by technological solutions to support collaboration, information sharing and performance management [31, 95]. Although change management approaches were not the focus of this review, the evidence identified in this study consistently indicated that effective change management is required in any efficiency improvement initiative.

## Discussion

We identified 82 studies which presented evidence of approaches, factors and considerations for managing efficiency improvement at a whole-of-system level and have synthesized this content to consolidate current evidence. A number of common public health system management approaches including continuous restructuring, delays in performance reporting, the setting of short-term reactive goals and the prioritization of cost reduction over service enhancement were identified as barriers to efficiency improvement [82]. The historical design of health systems based around siloes and service episodes were also flagged as barriers [96]. Despite the interlinked nature of financial performance and health services outcomes, these outcomes are not always addressed concurrently [49]. Capability, enthusiasm and centrally-led support for improvement initiatives across public health systems are not always sufficient to enable sustained results [2]. It was suggested that the efficiency benefits of technology are frequently overrated, as developing and implementing such solutions increases cost [40, 97].

While the approaches in this review vary in their setting across local and health system perspectives, this synthesis brings focus to the role of the system management entity in leading these approaches in order to support efficiency improvement. This review has identified that the current body of research is not cohesive and does not provide evidence of a best-practice standard for supporting efficiency improvement in public health systems. Rather, many individual factors were identified which collectively relate to public health system management approaches to improving efficiency; together these factors may inform the design of a comprehensive support framework.

## Implications and application

This study presents a synthesized view of current peer-reviewed evidence relevant to supporting efficiency improvement in public health systems. This paper addresses the gap in the literature in this space by outlining the range of current evidence-based factors and strategies associated with supporting both efficiency and service improvement in public health systems from the central system management perspective. These findings provide guidance on efficiency improvement approaches which are linked with success and may also be used to guide reflection on current practice, identify additional opportunities to explore and to validate approaches. Our review findings suggest that delaying essential activity and expenditure to represent short-term financial improvements is not recommended and can be detrimental to service quality, accessibility and stability [38, 46].

Evidence was identified in support of benchmarking, transparent target setting and timely progress reporting for efficiency improvement, while pairing financial improvement initiatives with efficiency improvement and strategic directions was also recommended [21, 88]. Sufficient resourcing and data access to support and monitor improvement initiatives is required in order to commence improvement projects [55]. Effective stakeholder engagement and consultation is an enabler of successful change [98]. Service integration, collaboration, locally-tailored solutions and knowledge sharing were clear themes in approaches that were considered successful in supporting efficiency improvement [26, 53, 69]. There was sound evidence for system-wide efficiency improvement activities to be led by central system management entities [63]. Specific teams and processes located at appropriate central decision-making levels within system management entities can lead efficiency improvement processes by monitoring efficiency improvement performance, enabling knowledge sharing, enhancing engagement with efficiency improvement and coordinating system-wide efficiency improvement programs [1, 2, 27].

## Limitations

Use of rapid review methodology was relevant to the broad and diverse literature in this field yet carries some limitations with potential for omission of relevant works. By searching only two databases in this study with minimal overlap in search results, the possibility that published work relevant to the review question may have been overlooked cannot be excluded. Similarly, by focusing only on published peer-reviewed works, relevant perspectives in other works may also have been excluded. While the search terms selected were broad and relevant, given the diverse and far-reaching nature of the topic, it is possible that relevant material may have been missed where this material was described using different key terms than those selected for this study.

## Conclusion

The requirement for efficiency in the delivery of public health services continues to increase. Addressing the challenges and enabling factors identified in this study represents an evidence-based approach for public health system management bodies to embed efficiency improvement. Combining these factors can inform a framework for supporting efficiency improvement in public health systems. By providing dedicated system-wide support for efficiency improvement, central health system management bodies can promote efficiency improvement in parallel with improved stakeholder and service quality outcomes.

## Supplementary Information

**Additional file 1. **

## Data Availability

All data generated or analysed during this study are included in this published article (and its supplementary information file).
